# Need for Distinctiveness Leads to Pathological Internet Use? The Perspective of Cognitive Behavioral Model

**DOI:** 10.3390/ijerph20021609

**Published:** 2023-01-16

**Authors:** Wei Zeng, Hua Wei, Meiting Liu

**Affiliations:** 1 Shen Jun Ru Law School, Hangzhou Normal University, Hangzhou 311121, China; 2 Normal College, Qingdao University, Qingdao 266071, China; 3 Faculty of Social Studies, University of Turku, 20500 Turku, Finland

**Keywords:** cognitive behavioral model of pathological Internet use, need for distinctiveness, maladaptive cognition, internet addiction, gender difference, gender role theory

## Abstract

Based on the cognitive behavioral model of pathological Internet use and the gender role theory, this present study investigated the association between the need for distinctiveness and pathological Internet use. Additionally, we explored a mediating role of maladaptive cognition in the association between the need for distinctiveness and pathological Internet use and tested whether the mediation model was moderated by gender. A sample of 745 Chinese university students (*M*_age_ = 19.92, *SD*_age_ = 1.42) was studied and participants completed anonymous questionnaires regarding the need for distinctiveness, maladaptive cognition, and pathological Internet use. Results revealed that the need for distinctiveness was positively associated with pathological Internet use, and the association between the need for distinctiveness and pathological Internet use was mediated by maladaptive cognition. In addition, gender moderated the association between maladaptive cognition and pathological Internet use; the effect was stronger for female participants than male participants. The findings expanded our understanding of the dark side of seeking distinctiveness. Practically, the results suggest that policymakers and psychological practitioners consider gender in preventing and intervening in pathological Internet use.

## 1. Introduction

Growing in size and complexity, the Internet has become increasingly significant in everyday life. Although the Internet has greatly improved the lifestyles of modern people, pathological Internet use (PIU) has become a salient concern in today’s society. Pathological Internet use (PIU) refers to a pathological state where individuals cannot control their time and strength spending on the Internet, which manifests as an obsession with the online content and the compulsion to linger on the Internet both physically and psychologically; such uncontrolled behavior may bring troubles to individual living, study, and work [1]. Previous researchers have given substantial attention to PIU, finding that Internet addiction is affected by both the individual characteristics (e.g., personality, self, cognition, emotion, need satisfaction) and environmental characteristics [2,3,4,5,6,7,8]. Despite considerable research, the effect of the need for distinctiveness, as an individual characteristic, on PIU, has rarely been examined.

The need for distinctiveness is a fundamental need for human beings and may greatly influence individual mental health [9,10,11]. Considering that PIU is an important indicator of mental health in a digitalized era, we wonder whether the need for distinctiveness is associated with PIU. Furthermore, the individual need for distinctiveness has dramatically increased, even in countries where traditional cultural norms do not favor a distinctive member [12,13,14]. Therefore, this present study aims at examining the association between the need for distinctiveness and PIU and to explore the potential influential mechanism underlying this association.

### 1.1. Need for Distinctiveness and PIU

The need for distinctiveness refers to a psychological need concerning individuals being particularly special or standing out from the crowd [15]. According to the anonymity, convenience, escape model (ACE model), the three features of the Internet including anonymity, convenience and escape, contribute to PIU [16]. This model has also been demonstrated by copious empirical studies [17,18,19,20,21]. Therefore, we assumed that individuals high in need for distinctiveness are more likely than individuals low in need for distinctiveness to satisfy their needs on the Internet, which may lead to PIU.

Of the three features, the anonymity of the Internet provides individuals with a safer place to present their distinctiveness. According to optimal distinctiveness theory, individuals always seek an optimal distinctiveness level, as distinctiveness above the threshold may bring them a negative evaluation, isolation, and even rejection from others during social interaction [10,22]. Concerning the potential risks, individuals would find it unpleasant to display distinctiveness in real-life situations and thus desire a relatively free and secure place to present distinctiveness. 

Additionally, anonymity makes the Internet such a place where people can make self-presentation without being negatively evaluated, isolated, and rejected [23,24,25]. Previous research has found that people prefer to deliver distinctive opinions through online communication than face-to-face interaction [23,24,25]. Students feel little humility or shame when answering questions online anonymously, because anonymity reduces evaluation apprehension, which is otherwise usually inevitable in an identified situation [25]. In such anonymous environments, individuals high in need for distinctiveness can be free from their real identities, having more freedom to choose the means and content for self-presentation [26]. 

Compared with seeking distinctiveness in reality, seeking it online is more convenient. Manifesting distinctiveness in real life usually involves external materials (e.g., hairstyle, clothes, decorations, names, etc.) [12]. In the online situation, however, individuals can use virtual products, which are more accessible and cheaper than the actual products themselves. For instance, individuals can customize their body features and clothes to show their distinctiveness in online games [27]. These costumes may cost less than their actual clothes or even free.

Moreover, individuals can escape the potential difficulties they may encounter when seeking distinctiveness in real life. The person–environment fit theory maintains that when a person is in conflict with their environment’s collective value, they may experience maladaptive problems and high pressure [28,29,30,31]. According to the person–environment fit theory, individuals who seek distinctiveness in real life may sometimes encounter social problems, which might cause them high pressure and negative emotions. In China, the individuals’ need for distinctiveness may collide with the cultural value of collectivism, which highlights the consistency of all members in a group [32]. Empirical evidence has also shown that, in Japan, where collectivism prevails, the individual need for distinctiveness is negatively correlated with their income, current life satisfaction, anticipated life satisfaction, and satisfaction with personal relationships [11]. Therefore, we suspect that in the Chinese context, individuals high in the need for distinctiveness is likely to have more pressure and mental issues than their counterparts. The difficult encounters in real life may push them toward finding another place to escape. 

Correspondingly, the Internet is an ideal place to escape as it is flooded with entertaining and stimulating content, immersing people and making them forget their troubles in real life for a short time [16]. In the short-term, Internet use buffers thwarted experience and negative emotions in real social life. As we have reasoned above, individuals high in the need for distinctiveness are very likely to encounter social pressure and mental issues in a collectivist context. When frustrated by these troubles in real life, individuals high in the need for distinctiveness are likely to be pulled into an online shelter. In the long-term, frequently escaping from existing problems may develop into habitual avoidant behavior, consequently worsening the adverse situation where these individuals have been struggling. 

In conclusion, the Internet is a suitable place for seeking and presenting distinctiveness, meeting people’s need for distinctiveness. Hereby, we propose the first hypothesis,

**Hypothesis** **(H1).** *The need for distinctiveness is positively associated with PIU.*

### 1.2. The Mediating Role of Maladaptive Cognition

Maladaptive cognition of the Internet is defined as distorted cognition of the self and the world [33]. Individuals high in the maladaptive cognition consider the online world to be much more comfortable than the offline world, and thus the self online is also more excellent than the self offline. Examples of measure items are “The Internet is the only place I am respected” and ‘‘I am worthless offline, but online I am someone”. Numerous studies from different countries have found a positive correlation between maladaptive cognition and PIU [3,34,35,36,37,38,39,40]. 

Davis’s cognitive behavioral model of problematic Internet use (PIU) states that PIU can be influenced by both proximal factors and distal factors, and distal factors function through proximal factors [33]. In this model, individual characteristics (e.g., depression, anxiety) are included as distal factors, and maladaptive cognition as a proximal factor. The model also argues that individual characteristics influence PIU through the mediation of maladaptive cognition [33,34,37,38]. Accordingly, we assumed that the need for distinctiveness as an individual characteristic is a distal factor that may influence PIU, and maladaptive cognition is a proximal factor and plays a mediating role in the association between the need for distinctiveness and maladaptive cognition. The specific reasoning is as follows. 

On one hand, the need for distinctiveness may be positively associated with maladaptive cognition. As the Internet bears the features of anonymity, convenience, and escape, it facilitates seeking and presenting distinctiveness and satisfies people’s need for distinctiveness. If individuals high in the need for distinctiveness compare the real world with the Internet world in terms of the satisfaction of psychological needs, they will easily form a distortedly positive view toward the Internet. Previous research has demonstrated that a personal sense of distinctiveness can promote the construction of self-identification and elevate self-evaluation [9,10]. Additionally, the Internet, compared with the real world, is likely to lead people to develop an excessively high evaluation of an Internet self. Given that over evaluations toward the Internet and a virtual self are key elements of maladaptive cognition [33], we suggest that the need for distinctiveness may increase maladaptive cognition.

On the other hand, maladaptive cognition might be positively associated with PIU. When the maladaptive cognition is activated, individuals will find it difficult to adapt to the real world and will be easily attracted by the virtual world. Subsequently, they may eventually become addicted to the Internet before spending too much time and effort on it [33]. Empirical research also indicated that maladaptive cognition can increase the risks of PIU [34,35,37,38,39,41].

Therefore, we propose our second hypothesis:

**Hypothesis** **(H2).** *Maladaptive cognition mediates the association between the need for distinctiveness and PIU; the need for distinctiveness is positively associated with maladaptive cognition, which is positively associated with PIU.*

### 1.3. The Moderating Role of Gender

Previous studies have found that gender moderates the association between the environmental and individual factors and PIU [42,43,44,45,46]. Compared with male Internet users, female Internet users are affected to a greater extent by these risk factors [44,46]. Drawing from gender role theory and previous studies, we assumed that gender moderates the association between maladaptive cognition and online addiction. Namely, women who have maladaptive cognition are more likely to develop PIU than men who have maladaptive cognition. 

Individuals high in maladaptive cognition would detest an offline social environment, think they are not loved in real life, and that people around them treat them badly [33]. These problematic thoughts may hinder the people’s satisfaction of the relatedness need [47]. The relatedness need is a basic human need and people may constantly seek fulfillment in another situation if their need for relatedness is unfulfilled in one given situation. As the Internet provides people with abundant convenience concerning socializing and interpersonal relationships, individuals who score high in maladaptive cognition would seek their unfulfilled relatedness need online. 

Although people generally need the satisfaction of relatedness, women are expected to be more attentive than men to the satisfaction of the relatedness need [48,49]. Gender role theory asserts that women and men are provided by parents and teachers with different values during socialization, which leads women to think more highly of the socializing value than men [50]. The focus on the socializing value may result in a situation where women feel more unbearable than men toward dislikes and social rejection, and thus women have a stronger motivation to seek vicarious satisfaction when encountering social frustration [51]. Correspondingly, women may pursue the satisfaction of their relatedness need on the Internet when they feel that they are not liked in real life. Thus, we assume that the effect of maladaptive cognition on PIU may be stronger for women than that for men. Hereby, we propose the third hypothesis:

**Hypothesis** **(H3).** *Gender moderates the indirect effect from maladaptive cognition to PIU in the association between the need for distinctiveness and PIU. Specifically, the indirect effect is much stronger for females than males.*

### 1.4. The Current Study

**Figure 1:**
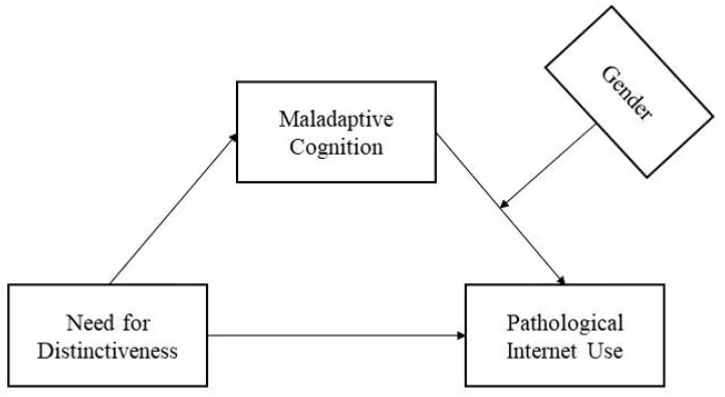
The structural model.

The principal aim of the current study was to test whether the need for distinctiveness was positively associated with PIU. In addition, we also aimed to test a moderated mediation model where maladaptive cognition mediates the association between the need for distinctiveness and PIU. Finally, gender moderated the effect of maladaptive cognition on PIU. The structural model is shown in Figure 1.

## 2. Methods

### 2.1. Participants 

This study recruited 766 college students from three universities in central China. Participants were informed of the principle of confidentiality and had the right to exit the investigation at any time. Twenty-one students either responded to questionnaires with regular answers or did not complete any of the items we measured. Finally, 745 college students (*M*_age_ = 19.92, *SD*_age_ = 1.42; 37.4% males) completed the whole set of questionnaires without missing data.

### 2.2. Measures 

#### 2.2.1. Need for Distinctiveness

Need for distinctiveness was measured by the Chinese version of a 5-point (1 = “totally disagree” to 5 = “totally agree”) self-attributed need for uniqueness scale including four items (e.g., I prefer being different from other people) [12,52]. This scale has been used among the Chinese population [12] with good reliability and validity. In this study, Cronbach’s alpha for the scale was 0.86.

#### 2.2.2. Maladaptive Cognition

The maladaptive cognition scale included four items [33,41] (e.g., I am worthless offline, but online I am someone). Answers were given on a 5-point Likert scale (1 = “never,” 5 = “always”). This scale has been widely used in previous research and has demonstrated good reliability and validity among Chinese samples [33,41]. In this study, Cronbach’s alpha for the scale was 0.86.

#### 2.2.3. PIU

The PIU was measured by the Chinese version of the PIU scale, which included eight items [53,54,55] (e.g., I repeatedly made unsuccessful efforts to control, cut back, or stop Internet use). Answers were given on a 6-point Likert scale (1 = “totally disagree,” 6 = “totally agree”). This scale has been widely used in previous research and has demonstrated good reliability and validity among Chinese samples [7,54]. In this study, Cronbach’s alpha for the scale was 0.87. 

### 2.3. Data Analysis

In this study, SPSS was used to analyze the data. A descriptive analysis was performed to examine the characteristics of the participants regarding the studied variables. A correlation analysis was performed to examine the correlations between each variable. In addition, the SPSS PROCESS macro was used to test whether maladaptive cognition could mediate the association between the need for distinctiveness and PIU. This SPSS PROCESS macro, suggested by Hayes (2013), was especially developed to test complex models with bootstrapping techniques calculating confidence intervals, which has been widely used in recent studies [56].

## 3. Results

### 3.1. Common Method Bias Test

It is known that using self-reporting to collect data might bring about common method bias. Thus, according to previous research, we attempted to limit any common method bias by surveying anonymously, but also used confirmatory factor analysis to test whether there was any significant common method bias [57]. The fit indices were as follows: *χ*^2^/df = 28.05, NFI = 0.49, RFI = 0.41, CFI = 0.50, IFI = 0.50, and RMSEA = 0.19. Therefore, there was no serious common method bias in the data of this study.

### 3.2. Preliminary Analysis

Pearson correlations as well as the means and standard deviations of all the study variables are presented in Table 1. The need for distinctiveness was positively correlated with maladaptive cognition and PIU; maladaptive cognition was positively correlated with PIU.

### 3.3. Testing for the Moderated Mediation Model

**Figure 2:**
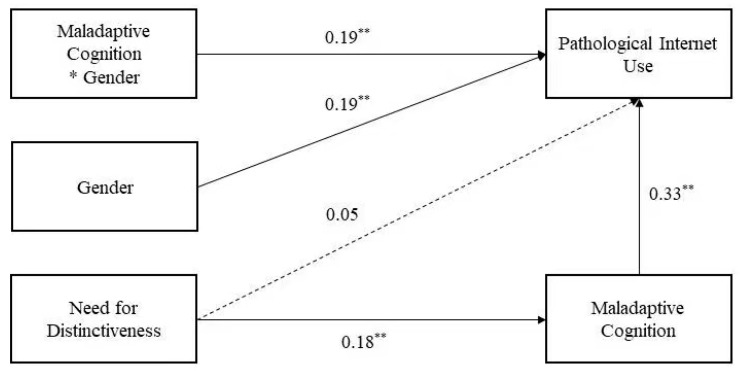
The confirmed structural model. * *p* < 0.05; ** *p* < 0.01.

Multiple regressions were conducted to test the mediated moderation model, and the results are shown in Table 2. According to prior studies [56], we first conducted Model 4 in SPSS PROCESS macro to test whether maladaptive cognition mediated the effect of the need for distinctiveness on PIU. Results revealed that the need for distinctiveness positively predicted maladaptive cognition (*B* = 0.18, *p* < 0.01) (Model 1). The need for distinctiveness could not predict PIU (*B* = 0.05, *p* > 0.05), maladaptive cognition positively predicted PIU (*B* = 0.42, *p* < 0.01) (Model 2), and the mediating effect was 0.07, *SE* = 0.02, 95% CI *=* [0.04, 0.11]. These results indicate that maladaptive cognition mediated the effect of the need for distinctiveness on PIU. The moderated mediation analysis via the PROCESS macro (Model 14) showed a significant interaction of maladaptive cognition and gender (*B* = 0.19, *p* < 0.01) (Model 3). These results indicate that the association between the need for distinctiveness and PIU was moderated by gender. The confirmed structural model is illustrated in Figure 2. 

**Figure 3:**
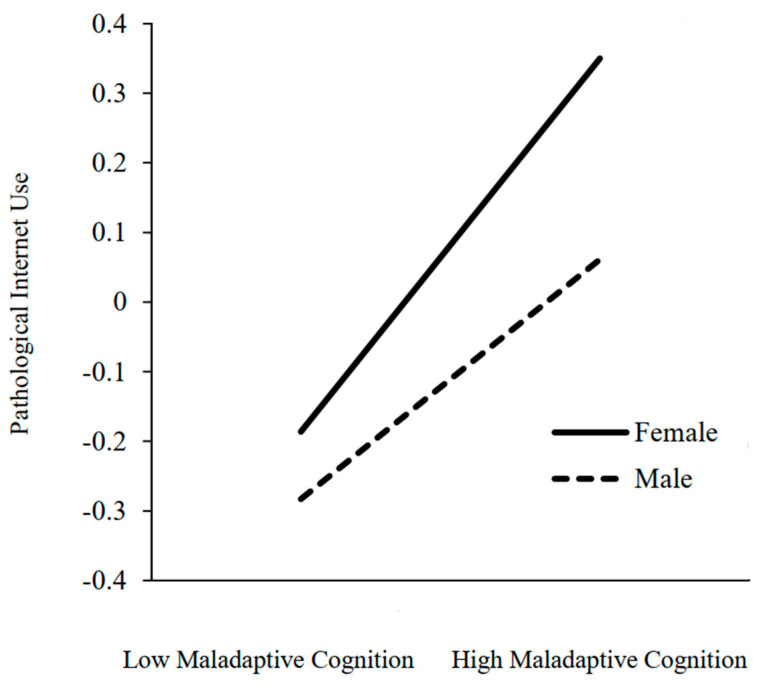
The interaction of maladaptive cognition and gender on PIU.

As shown in Figure 3, simple slope tests showed that for male participants, maladaptive cognition was significantly associated with PIU (simple slope = 0.33, *t* = 7.15, *p* < 0.01). However, for female participants, the association of maladaptive cognition with PIU remained significant but stronger (simple slope = 0.52, *t* = 10.92, *p* < 0.01). Conditional indirect effects revealed that, for male participants, the indirect effect was 0.06, 95% CI *=* [0.03, 0.09]; for female participants, the indirect effect was 0.09, *SE* = 0.02, 95% CI = [0.05, 0.14], and the index of the moderated mediation was 0.03, *SE* = 0.02, 95% CI *=* [0.08, 0.64]. Namely, the indirect effect of the need for distinctiveness on PIU through maladaptive cognition was stronger for females than males. These results indicate that the mediating effect of the need for distinctiveness on PIU through maladaptive cognition is moderated by gender.

## 4. Discussion

This study examined the association between the need for distinctiveness and PIU and tested the mediating effect of maladaptive cognition and the moderating effect of gender. To our knowledge, this present study is the first to investigate the association between the need for distinctiveness and PIU and the functioning mechanism. 

### 4.1. The Need for Distinctiveness and PIU

The results of the study showed that individuals high in the need for distinctiveness are more likely to develop PIU. On one hand, the results draw more academic attention to the subtle risk factors that lead to PIU. Previous research has focused on the effect of explicit negative environmental and individual characteristics (e.g., hash parenting, deviant peer interaction, social anxiety, and impulsivity) on PIU [34,54,58]. Neutral factors or even positive factors in Western culture are relatively neglected. For example, distinctiveness is a social value widely recognized in mainstream Western culture. It is also considered to be a positive individual characteristic and is essential in constructing one’s self-identity and boosting self-esteem. Empirical evidence has shown that a personal sense of distinctiveness is positively associated with optimism, hope, resilience, and positive self-evaluation [9], and it helps to enhance the individual psychological state [59]. Despite its widely accepted advantages, distinctiveness may increase the risk of PIU according to our current results. 

On the other hand, the increasing need for distinctiveness across the world suggests that examining the effect of need for distinctiveness on PIU is significant. Recent data have shown that the need for distinctiveness among the Chinese has been increasing from 1950 to 2000 [12]. Growing numbers of American parents gave their children uncommon names spanning from 1880 to 2007 [14]; Japanese parents were also inclined to give their children uncommon names from 2004 to 2013 [13]. Given a close link between individualism and the need for distinctiveness, the prevalence of individualism across the world [60] seems to signify a global increase in the need for distinctiveness. 

### 4.2. The Mediating Role of Maladaptive Cognition

The results further enrich the cognitive behavioral model of PIU. This study revealed that the need for distinctiveness as a “positive” individual character in individualist culture [22] can be positively associated with PIU through the mediation of maladaptive cognition in a collectivism culture. This result can be explained by the person–environment fit theory, which maintains that the match between the characteristics of a person and the person’s environment predicts a variety of positive outcomes including satisfaction, performance, and self-esteem [29,30,31]. Seeking distinctiveness involves an individual standing out from the group and manifesting distinctive characteristics. 

Although the need for distinctiveness fits in the individualistic value, collectivist culture usually disdains such attitudes and behavior because integration, dependence, and conformity are much more highlighted within collectivism. As an ancient Chinese saying goes, “it is the taller trees in the woods that get their tops blown off”. This means that an outstanding person is liable to be attacked. Empirically, research has found that standing out from a group is a sign of selfishness in the East Asian cultural context [61]. Therefore, seeking distinctiveness in China is likely to receive social punishment and encounter adaptive issues such as anxiety and depression, thereby increasing the risks of maladaptive cognition and PIU [33].

In the extant research on PIU, very few of them chose factors bearing cultural particularity. Previous scholars have merely investigated the individual characteristics (social anxiety, psychological distress, psychoticism, and impulsivity) without prominently including cultural particularity. These individual characteristics are almost negative in any given context. However, the need for distinctiveness is more positive in an individualistic culture than in a collectivist culture. Therefore, the current results may not hold for people from individualistic cultural backgrounds. From the perspective of the person–environment fit, it is the mismatch between the individual and their environment that induces maladaptive cognition, which eventually develops into PIU, rather than the individual characteristics themselves. Therefore, future research can examine the cognitive behavioral model of PIU from the perspective of the person–environment fit.

### 4.3. The Moderating Role of Gender

This study showed that gender moderates the association between maladaptive cognition and PIU. Compared with men, women are more likely to be addicted to the Internet when experiencing maladaptive cognition. We explained the result by the gender difference of evaluating the socializing value. In addition, the gender difference in the influence of maladaptive cognition on PIU can also be explained from the gender difference in personality. Previous studies have found that women have higher levels of social anxiety and shyness than men [62,63]. Individuals high in social anxiety and shyness prefer the anonymity of the Internet, and are more vulnerable to PIU [64,65]. Therefore, we theorize that when maladaptive cognition causes an individual to use the Internet, women may use it more than men, and are more likely to become addicted to it.

The results of our study support previous studies on the gender difference in the association between risk factors and PIU. Compared with males, female users with PIU are more easily affected by risk factors. For example, the association between ADHD and PIU was stronger among female than male college students [46]. Perceived paternal Internet use behavior can significantly predict female PIU but not male PIU [44]. The association between peer victimization and mobile phone addiction [66] is also stronger among girls than boys. 

Although previous studies have investigated the gender difference of PIU regarding attention, emotion, and interpersonal risk factors, few studies have examined the gender difference in the association between cognitive risk factors (e.g., maladaptive cognition) and PIU. 

The results of this study deepen the understanding of the cognitive behavioral model of PIU. As individual difference is barely discussed in the original cognitive behavioral model of PIU [33], the results of the present study expand the theoretical model. Therefore, future research can continue to investigate whether there are other individual factors that mediate the association between maladaptive cognition and PIU. Practically, there are intervention programs for PIU that focus on changing the patients’ maladaptive cognition. The results of this study suggest that we pay particular attention to the maladaptive cognition of female Internet addicts. 

### 4.4. Limitations and Implications

To begin with, the use of a convenient sample limits the generalization of the findings. As the participants were university students, who are more likely to freely use the Internet and therefore at more risk of being addicted to the Internet than individuals in other age groups, the results should be tested among broader groups in the future. Moreover, the results of our study may be limited in cultural generalization because only participants from mainland China were recruited. Compared to Western countries, China is still a collectivist-oriented country where social relations are heavily underlined. In China, individuals who pursued distinctiveness might experience interpersonal hardships in daily life [12]. Such cultural particularity in China might strengthen the effect of need for distinctiveness on PIU. Therefore, future research can investigate the association between need for distinctiveness and PIU in other cultural contexts and test whether the present results can be obtained.

In addition, the current study merely discussed the investigated maladaptive cognition as the underlying mechanism between the need for distinctiveness and PIU. We presume that there are other mechanisms that deserve to be examined in the future. For instance, identity confusion may be a potential mediator. Erikson’s theory of psychosocial development argues that adolescents commit themselves into building and developing their self-identity at the identity versus role confusion stage [8,67]. When the personal needs collide with what the social environment requires, young people may develop identity confusion. In China, where group conformity is highly regarded, young people may encounter social obstacles on the road to satisfying their need for distinctiveness. Under these circumstances, individuals who have a higher need for distinctiveness may experience stronger collision between their personal needs and group needs, leading to identity confusion. Young people high in identity confusion tend to conduct identity experiments on the Internet [68], during which they may develop PIU [69].

Some practical implications can be concluded from this study. First, educators can create an open-minded, tolerant environment on campus to meet the students’ need for distinctiveness. If their needs are gratified in an offline social world, seeking ways to satisfy their relatedness need online will be unnecessary. For example, schools and universities can offer students more freedom to outfits and courses and organize activities where students can manifest their uniqueness. Additionally, psychological counseling practitioners can intervene in PIU using cognitive behavioral therapy, an effective series of procedures of intervening PIU through altering the clients’ cognition [70,71]. Given that the current study revealed maladaptive cognition as the mediator on the relationship between need for distinctiveness and PIU, adopting cognitive behavioral therapy to intervene maladaptive cognition can be another direction.

When engaging in the intervention of PIU among college students, practitioners should pay more attention to female students. Our current study found that for female college students, maladaptive cognition is a strong predictor of PIU than for male college students, which suggests that intervening in the PIU of female college students by intervening in their maladaptive cognition can be more effective than practicing it on male college students. Moreover, helping female students break through the traditional gender norm may decrease the odds of developing PIU. Practitioners and educators can decrease the PIU of female students by guiding them to recognize the established, but not necessarily required, gender concepts during individual development. Specifically, previous research on mental health indicated that women who rated themselves high in femininity tended to have more mental issues while those who were more androgynous, meaning having nearly comparable portions of femininity and masculinity, had better mental conditions than their counterparts [72]. Correspondingly, helping female students build a flexible concept of an androgynous social gender by introducing some traits that are traditionally regarded to be exclusive in men, for instance, being active, decisive, and persistent, can protect female students from PIU.

## 5. Conclusions

To sum up, the present study enriches our understanding of the risk factors contributing to PIU and the mechanisms linking the need for distinctiveness to PIU among Chinese university students. Maladaptive cognition mediates the need for distinctiveness and PIU, and gender moderated the association between maladaptive cognition and PIU. The mediating role of maladaptive cognition and the moderating role of gender together contribute to uncovering how the need for distinctiveness is associated with PIU and provide insights into intervening in the PIU of college students.

## Figures and Tables

**Table 1:** Correlation coefficients of the variables.

Variables	*M*	*SD*	1	2	3	4	5
1. Age	19.92	1.42	—				
2. Gender	0.63	0.48	−0.08 *	—			
3. Need for distinctiveness	2.89	0.84	0.02	−0.10 **	—		
4. Maladaptive cognition	1.25	0.48	0.03	−0.09 *	0.18 **	—	
5. PIU	2.25	0.96	0.01	0.05	0.12 **	0.43 **	—

Note: * *p* < 0.05; ** *p* < 0.01.

**Table 2:** Multiple regression analyses.

	Model 1 (Criterion = MC)		Model 2 (Criterion = PIU)		Model 3 (Criterion = PIU)	
Predictors	*B*	*SE*	*B*	*SE*	*B*	*SE*
NFD	0.18 **	0.04	0.05	0.03	0.05	0.03
MC			0.42 **	0.03	0.33 **	0.05
Gender					0.19 **	0.07
MC × Gender					0.19 **	0.07
*R* ^2^	0.03		0.18		0.20	
*F*	23.35 **		83.61 **		46.66 **	

Note: NFD = need for distinctiveness, MC = maladaptive cognition, PIU = pathological Internet use. * *p* < 0.05; ** *p* < 0.01.

## Data Availability

The datasets generated during and/or analyzed during the current study are available from the corresponding author on reasonable request.
